# Acute-Onset Lower Extremity Weakness and Urinary Retention in a Chronically Immunosuppressed Patient: Diagnosis and Management of Herpes Simplex Virus Type 2 Myelitis

**DOI:** 10.7759/cureus.55248

**Published:** 2024-02-29

**Authors:** Jaya Chandra, Hamid R Khatibi

**Affiliations:** 1 Internal Medicine, Mercy Health St. Rita's Medical Center, Lima, USA

**Keywords:** chancre, bilateral lower extremity weakness, acute urinary retention (aur), immunosuppressed, myelitis, herpes simplex virus type 2 (hsv-2)

## Abstract

A 34-year-old immunosuppressed male presented with worsening bilateral lower extremity weakness and urinary retention accompanied by a painless clean-based chancre on his glans penis. Physical examination revealed symmetrically diminished lower extremity weakness most pronounced with hip flexion and knee extension and absent Achilles reflexes. Full MRI spine without contrast was noncontributory. Lumbar puncture showed elevated protein and total nucleated cells with lymphocytic predominance. Both CSF and serum polymerase chain reaction were positive for herpes simplex virus type 2. He received IV methylprednisolone and acyclovir and underwent four months of physical therapy with complete resolution of his neurologic deficits.

## Introduction

Ascending motor weakness and urinary retention can raise higher clinical suspicion for a demyelinating process such as acute inflammatory demyelinating polyneuropathy [1]. However, a comprehensive evaluation of recent illness, sexual history, immunosuppression status, and associated dermatological findings can broaden the differential diagnoses. The timeline of different symptoms may also aid in narrowing the differentials and may prompt further investigation into certain aspects of a patient’s medical history. Herpes simplex virus type 2 (HSV-2) infection can cause sensory symptoms such as paresthesia, motor weakness or flaccid paresis, or autonomic dysfunction. Dermatological manifestations, such as herpetic eruptions, may not always occur with the onset of neurological symptoms [2]. In this report, we present a chronically immunosuppressed patient who developed worsening bilateral ascending lower extremity weakness with urinary retention and was initially thought to have acute inflammatory demyelinating polyneuropathy at an outside facility prior to being transferred to our hospital. On further evaluation during foley exchange, the patient was noted to have a painless clean-based chancre on his glans penis which prompted additional diagnostic workup for sexually transmitted diseases, specifically herpes simplex virus (HSV). This case demonstrates the importance of obtaining a comprehensive history and physical examination.

## Case presentation

**Figure 1:**
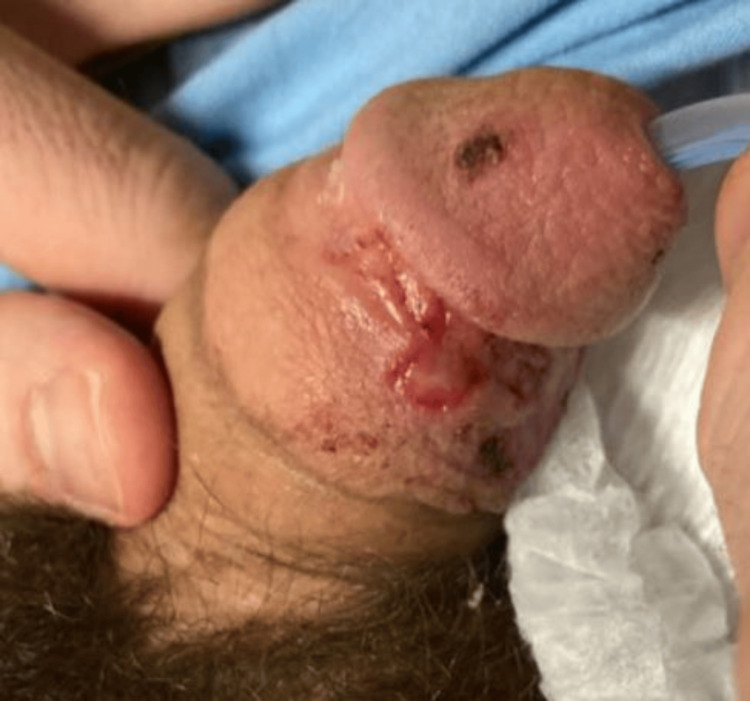
Painless clean-based chancre on the glans penis

A 34-year-old male status post renal transplant for medullary sponge kidney disease on an anti-graft rejection regimen (mycophenolate and tacrolimus) initially presented to an outside hospital with a three-week history of progressively worsening bilateral lower extremity weakness and urinary retention requiring foley catheter placement. On further evaluation, he was noted to have a painless clean-based chancre on his glans penis, which he attributed to traumatic foley placement (Figure 1).

Furthermore, he reported an associated upper respiratory viral illness approximately one week prior to the onset of lower extremity weakness and dysuria approximately three weeks prior which had spontaneously resolved. Physical examination revealed symmetrically diminished lower extremity weakness most pronounced with hip flexion and knee extension and absent Achilles reflexes (Video 1).

**Video 1:** Bilateral lower extremity deficits on physical examination Recording of bilateral lower extremity deficits on physical examination demonstrating symmetrically diminished lower extremity weakness most pronounced with hip flexion and knee extension and absent Achilles reflexes.

Full MRI spine without contrast was non-contributory. The patient denied a history of sexually transmitted infections and stated he was in a monogamous heterosexual relationship. Lumbar puncture showed elevated protein and total nucleated cells with lymphocytic predominance (Table 1). Both CSF and serum polymerase chain reaction (PCR) were positive for HSV-2 (Table 1).

**Table 1:** Serum, urine, and cerebrospinal fluid laboratory studies HSV: Herpes simplex virus; HIV: Human immunodeficiency virus; Ag: Antigen; Ab: Antibody; RPR: Rapid plasma reagin; CMV: Cytomegalovirus; IgM: Immunoglobulin M; IgG: Immunoglobulin G; EBV: Epstein-Barr virus; PCR: Polymerase chain reaction; CSF: Cerebrospinal fluid; HHV: Human herpes virus; VDRL: Venereal disease research laboratory test

Test	Observed value	Reference range
Serum studies		
HSV 1	Not detected	Not detected
HSV 2	Detected	Not detected
HIV Ag/Ab	Nonreactive	Nonreactive
RPR	Nonreactive	Nonreactive
Borrelia burgdorferi antibodies, IgM	Negative	Negative
CMV IgM	12.0	≤29.9 AU/mL
EBV Ab to viral capsid Ag IgG	>750.0	0.0-21.9 U/mL
EBV Ab to viral capsid Ag IgM	27.3	0.0-43.9 U/mL
EBV Ab to nuclear Ag IgG	169.0	0.0-21.9 U/mL
EBV Ab to early (D) Ag IgG	<5.0	0.0-10.9 U/mL
Parvovirus B19 Ab, IgG	3.99	≤0.90 IV
Parvovirus B19 Ab, IgM	0.19	≤0.90 IV
Urine studies		
Chlamydia trachomatis PCR, urine	Not detected	Not detected
Neisseria gonorrhea PCR, urine	Not detected	Not detected
CSF fluid analysis		
Glucose	75 mg/dL	40-80 mg/dL
Protein	531 mg/dL	12-60 mg/dL
Total nucleated cells	1963/cumm	0-5/cumm
Red blood cells	8000	0/cumm/cumm
Segmented cells	1%	0-6%
Lymphocytes	91%	0-90%
Monocytes	8%	0-45%
Culture	No growth	No growth
Gram stain	Rare segmented neutrophils were observed, no bacteria were seen	
CSF meningitis/encephalitis molecular panel		
Escherichia coli K1	Not detected	Not detected
Haemophilus influenza	Not detected	Not detected
Listeria monocytogenes	Not detected	Not detected
Neisseria meningitidis	Not detected	Not detected
Streptococcus agalactiae	Not detected	Not detected
Streptococcus pneumoniae	Not detected	Not detected
Cytomegalovirus	Not detected	Not detected
Enterovirus	Not detected	Not detected
HSV-1	Not detected	Not detected
HSV-2	Detected	Not detected
HHV-6	Not detected	Not detected
Parechovirus	Not detected	Not detected
Varicella-zoster virus	Not detected	Not detected
Cryptococcus neoformans/gatti	Not detected	Not detected
Additional CSF studies		
VDRL	Nonreactive	Nonreactive
Lyme antibody	0.92 LIV	≤ 0.99 LIV
West Nile antibody, IgM	0.53 IV	≤ 0.89 IV
West Nile antibody, IgG	0.14 IV	≤ 1.29 IV

The patient's partner later reported she had not been monogamous and had sexual intercourse with another person. She was found to be positive for HSV-2 and was determined to be the source of the patient's infection. The patient was diagnosed with myelitis and neurogenic bladder secondary to HSV-2 infection and received IV methylprednisolone 500 mg every 12 hours for three days and IV acyclovir 650 mg (10 mg/kg) every eight hours for 21 days. The patient’s clinical status gradually improved at the time of discharge to the inpatient rehabilitation unit. He was transitioned to life-long suppressive treatment with oral acyclovir 400 mg every 12 hours. The patient was ultimately discharged home after 19 days of inpatient rehabilitation with plans for outpatient physical therapy three times per week. Four months later, he was able to independently perform his activities of daily living without assistance and return to his regular exercise routine.

## Discussion

HSV-2 is a neurotropic virus that can reactivate in the sacral ganglia, dorsal root ganglia, and trigeminal ganglia and result in genital herpes, transient bladder paralysis, encephalitis, aseptic meningitis, myelitis, radiculopathy, and acute retinal necrosis [3,4]. HSV-2 has been found to cause thoracic or lumbosacral ascending myelitis, especially in immunocompromised patients. Sensory symptoms can include loss of sensation, ascending paresthesia, or back pain at or proximal to the level of myelitis. Motor symptoms include sphincter dysfunction and weakness or flaccid paresis of the arm and leg muscles, in particular the flexors of the lower extremities and extensors of the upper extremities. Autonomic dysfunction can present as constipation, urinary retention, temperature dysregulation, or episodes of hypertension [4]. Acute ascending HSV-2 myelitis often initially presents with urinary dysfunction and sensorimotor deficits in the lower extremities followed by progressively worsening ascending myelopathy [2]. MRI of the spine may demonstrate enlargement of the conus medullaris or lower spinal cord along with hyperintensity on T2-weighted images and enhancement of the adjacent roots on gadolinium-enhanced images [4].

Although the dermatological findings in combination with the sensorimotor deficits help in the diagnosis of HSV-2 myelitis, herpetic eruptions may not always be present with the onset of neurological symptoms. A lumbar puncture with PCR analysis of the CSF can help further guide the diagnosis. Although HSV is not commonly isolated from the CSF and HSV antibody titers may not be elevated early in the disease stage, PCR techniques have allowed for rapid, sensitive, and noninvasive diagnosis of HSV myelitis [2]. Quantitative measurements of CSF antibodies are not recommended in an acute diagnosis but can be helpful in retrospective diagnosis or if CSF is obtained later in the disease course and is PCR negative. Research has shown that PCR testing has a sensitivity and specificity of 98% and 94%, respectively, and positive and negative predictive values of 95% and 98%, respectively. Prompt treatment of HSV-2 myelitis should be initiated with acyclovir 10 mg/kg of body weight every eight hours for 14 to 21 days to prevent further progression of the disease [5].

Genital herpes lesions often present as painful skin lesions approximately 2 mm to 4 mm in size associated with surrounding erythema. Lesions can progress to ulcerations, erosions, or vesiculopustules. Vesicles and pustules may have a central umbilicated depression while ulcerations and erosions typically have scalloped borders. If active genital lesions are present in HSV-2 infections, vesicles can be unroofed to obtain viral cultures of vesicular fluid. Specimens can also be swabbed and tested via PCR or direct fluorescent antibody testing. Tzanck smear is an alternative form of testing via scraping of active genital lesions, but this method has low sensitivity and specificity and is not used as commonly as other types of tests. Research has shown that real-time PCR is a rapid, reproducible, and efficient diagnostic method for detecting HSV-2 in active genital lesions [6-9].

## Conclusions

In conclusion, HSV-2 can present with ascending myelitis, autonomic dysfunction, and dermatologic findings as seen in this patient with bilateral ascending lower extremity weakness, urinary retention, and painless chancre of the glans penis. Although the patient reported being in a monogamous heterosexual relationship, it was later determined that his sexual partner had not been monogamous. Despite the unremarkable MRI findings in this patient, the combination of his physical examination findings, timeline of symptoms, and CSF analysis helped to determine the final diagnosis. This case demonstrates that a comprehensive medical history should be obtained from patients as it can help with narrowing the differential diagnosis and guide further diagnostic evaluation.
